# A Facile Approach for Fabrication of Core-Shell Magnetic Molecularly Imprinted Nanospheres towards Hypericin

**DOI:** 10.3390/polym9040135

**Published:** 2017-04-07

**Authors:** Wenxia Cheng, Fengfeng Fan, Ying Zhang, Zhichao Pei, Wenji Wang, Yuxin Pei

**Affiliations:** Shaanxi Key Laboratory of Natural Products & Chemical Biology, College of Chemistry & Pharmacy, Northwest A&F University, Yangling 712100, China; 18829784980@163.com (W.C.); fanfengfeng1314@163.com (F.F.); teddyzhangy@163.com (Y.Z.); peizc@nwafu.edu.cn (Z.P.); wjwang@nwsuaf.edu.cn (W.W.)

**Keywords:** surface molecular imprinting, hypericin, magnetic nanospheres, dopamine, core-shell

## Abstract

By taking advantage of the self-polymerization of dopamine on the surface of magnetic nanospheres in weak alkaline Tris-HCl buffer solution, a facile approach was established to fabricate core-shell magnetic molecularly imprinted nanospheres towards hypericin (Fe_3_O_4_@PDA/Hyp NSs), via a surface molecular imprinting technique. The Fe_3_O_4_@PDA/Hyp NSs were characterized by FTIR, TEM, DLS, and BET methods, respectively. The reaction conditions for adsorption capacity and selectivity towards hypericin were optimized, and the Fe_3_O_4_@PDA/Hyp NSs synthesized under the optimized conditions showed a high adsorption capacity (*Q* = 18.28 mg/g) towards hypericin. The selectivity factors of Fe_3_O_4_@PDA/Hyp NSs were about 1.92 and 3.55 towards protohypericin and emodin, respectively. In addition, the approach established in this work showed good reproducibility for fabrication of Fe_3_O_4_@PDA/Hyp.

## 1. Introduction

Hypericin (Hyp) is one of the principal bioactive components of the St. John’s Wort plant (*Hypericum perforatum*). Traditionally used as anti-bacterial, anti-inflammatory, anti-depressive, and anti-virus agents, hypericin has gained increasing interest recently due to its potential as a highly effective anti-tumor photosensitizer [1]. Though hypericin can be obtained via chemical synthesis [2], extraction from the plant itself is still an indispensable supply pathway. However, hypericin exists in a very low concentration with structural similar analogs (such as pseudohypericin) in the plant [3]. This, together with its poor solubility and degradation upon exposure to heat and light, leaves its enrichment and purification from the plant a great challenge. This greatly inhibits the wide applications of hypericin in pharmaceutical fields. Establishing a facile method for producing efficient separation sorbents with high specificity and adsorption capacity towards hypericin is of significance and much desired [4].

Molecular imprinting [5,6] is a well-known modern technology for the production of nanostructured materials capable of molecular recognition, with high selectivity and good chemical stability at a low cost. Surface molecularly imprinted polymers (SMIPs) [7,8] are synthesized by allowing polymerization to take place on substrate surface, in order to create recognition sites on/near the material surface. These recognition sites, alongside the MIPs advantages mentioned above, provide faster rebinding kinetics for the target molecules, and are effective for the separation and enrichment of natural bio-compounds, making this an attractive solution to the challenge [9]. A variety of materials, including graphene [10], carbon nanotubes [11], silicon [12], silica particles [13], magnetic nanoparticles [14,15], inorganic material, and chips [16], have been used as substrates for construction of SMIPs. Among these, magnetic nanospheres (MNSs) are one of the most popular substrates for the synthesis of core-shell structured SMIPs [17]. Besides possessing advantages, such as high surface-to-volume ratio, low cost, regular shape with controllable size, and good mechanical stability, more significantly, their superparamagnetic property endows the final SMIPs facile magnetic response separation. This translates to potential applications in bio-imaging, drug delivery and therapeutics where the template is a drug [18], such as hypericin. However, to the best of our knowledge, at present there are no reports of core-shell structured SMIP nanospheres based on MNSs for specific recognition of hypericin.

Traditionally, the MIP layer (shell) around MNS (core) is formed by self-assembly of functional monomers and templates, followed by copolymerization with cross-linkers. The copolymerization is normally initiated by heat or UV light, and performed in organic solvents, with the process extending over a prolonged period of time (overnight or longer) [19]. Considering the increasing likelihood of hypericin to degrade during exposure to heat/light, it is obvious that the traditional procedure is not suitable for fabricating SMIPs for hypericin. Contrarily, dopamine can be oxidized and spontaneously self-polymerize, with oxygen as the oxidant under weak alkaline solution to yield polydopamine (PDA) [20]. Though the molecular mechanism behind the formation of PDA is complicated and not well-understood, the self-polymerization of dopamine is very mild and the cross-linked PDA network can adhere to virtually any type of material surfaces. These unique features make dopamine a promising functional monomer for fabrication of core-shell structured SMIP nanoparticles without a cross-linker (also called bi- or multi-functional comonomer), which is generally required. In fact, a few SMIP nanoparticles that were successfully prepared using dopamine as a monomer have been reported [14,21], including Fe_3_O_4_@PDA NPs as a stationary phase for recognition of OT-CEC [15] and SiO_2_@PDA nanoparticles for protein recognition and separation [13].

**Scheme 1:**
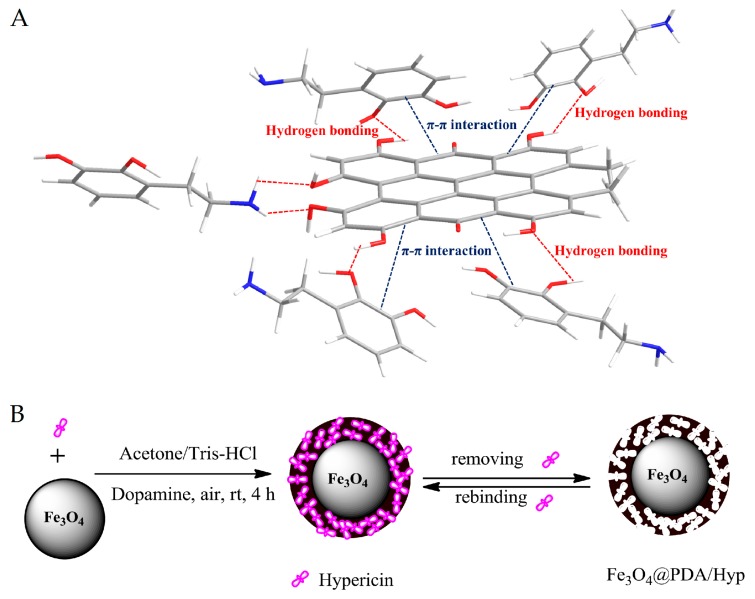
(**A**) Illustration of noncovalent bonding of template, hypericin with the functional monomer, dopamine: hydrogen bonding and π−π interaction; (**B**) Schematic illustration of fabricating Fe_3_O_4_@PDA/Hyp.

Inspired by the above-mentioned works, we envisioned that self-polymerization of dopamine on the surface of MNSs would provide a facile approach for fabrication of core-shell structured magnetic molecularly imprinted nanospheres for selective recognition of hypericin (Fe_3_O_4_@PDA/Hyp NSs). Herein, we investigated the feasibility for fabrication of Fe_3_O_4_@PDA/Hyp NSs by using dopamine as the only functional monomer. As depicted in Scheme 1, MNSs suspended in weak alkaline Tris-HCl buffer solution was first mixed with a solution of hypericin in acetone, followed by the addition of dopamine. Dopamine self-polymerized on the surface of MNSs in the air to form a thin layer of PDA within which hypericin molecules were embedded via non-covalent hydrogen bonding and π–π interactions. Removal of hypericin from the PDA layer leaves the recognition sites behind, and Fe_3_O_4_@PDA/Hyp NSs were thus obtained. The recognition properties of so-prepared Fe_3_O_4_@PDA/Hyp NSs toward hypericin were then evaluated and screened by varying reaction conditions.

## 2. Experimental Section

### 2.1. Chemicals and Instrumentation

Dopamine hydrochloride (DA, 98%), polyethylene glycol 1000 (PEG 1000), and ethanolamine were purchased from Aladdin, Shanghai, China. FeCl_3_·6H_2_O, ammonium hydroxide (28%), and ethylene glycol were purchased from Xilong Chemical Industry, Sichuan, China. Emodin was purchased from Xi’an Tianfeng Biological Technology Co., Ltd. (Xi’an, China) Acetone was purchased from Rionlon Bohua Pharmaceutical & Chemical Co., Tianjin, China. Anhydrous sodium acetate was purchased from Tianjin Bodi Chemical Industry Co., Ltd., Tianjin, China. Protohypericin (Protohyp) and hypericin (Hyp) were synthesized according to the procedures developed in our lab [22], and characterized by ^1^H-NMR (See Figures S1–S3).

NMR spectra were recorded on a Bruker 500 MHz Spectrometer (Bruker, Fällanden, Switzerland) with working frequencies of 500 MHz for ^1^H in DMSO-*d*_6_ or MeOD-*d*_4_. The residual signals from DMSO-*d*_6_ (^1^H: δ 2.50 ppm), or MeOD-*d*_4_ (^1^H: δ 3.31 ppm) were used as internal standards. Transmission Electron Microscope (TEM) images were taken by an H-600 instrument (Hitachi Ltd., Tokyo, Japan, 80 kV). The samples were prepared by dropping a droplet of the sample solution onto a TEM grid (copper grid, 300 meshes, coated with carbon film). Dynamic light scattering (DLS) measurements were performed on a DelsaTM Nano system (Beckman Coulter, Brea, CA, USA). UV-Vis spectra were recorded with Shimadzu 1750 UV-Visible spectrophotometer (Shimadzu, Tokyo, Japan) at 298 K. The surface area and the porosity of the prepared NSs were measured by nitrogen physisorption (Autosorb-iQ, Quantachrome, Boynton Beach, FL, USA), based on the Brunauer–Emmet–Teller (BET) method (ASAP 2020, Micromeritics Inc., Norcross, GA, USA). Samples were vacuum-degassed at 50 °C for 9 h before the adsorption experiments.

### 2.2. Preparation of Fe_3_O_4_ Magnetic Nanospheres (MNSs)

The MNSs were synthesized through the solvothermal approach according to the literature [23]. Briefly, 1.68 g FeCl_3_·6H_2_O was dissolved in 50 mL ethylene glycol with vigorous stirring until the solid was dissolved, then 4.5 g anhydrous sodium acetate and 1.25 g PEG were added to the solution, with continuous stirring for one hour. The resultant mixture was transferred into a Teflon-lined stainless steel autoclave (with a volume of 100 mL) and placed in an oven at 200 °C for 10 h, then cooled to room temperature. The obtained precipitate was washed with ethanol and deionized water several times and collected by magnet. The final product was dispersed in ethanol for further use.

### 2.3. Preparation of Hypericin-Imprinted Nanospheres (Fe_3_O_4_@PDA/Hyp) and Non-Imprinted Nanospheres (Fe_3_O_4_@PDA) 

A typical procedure for preparation of Fe_3_O_4_@PDA/Hyp NSs was as following: 25 mg MNSs were dispersed in 10 mM Tris-HCl solution (pH = 8.0 unless specified) by ultrasonication for 10 min. Then hypericin dissolved in acetone was added to the suspension by mechanically stirring for 10 min, followed by the addition of dopamine. The mixture was stirred under air with a mechanical stirrer for 4 h. The solid was collected by magnetic separation and washed first with ultrapure water several times, then alternately with an acetone solution containing acetic acid (3% in volume), and ammonium hydroxide (3% in volume), to remove the embedded template, until no hypericin in the supernatant was detected using UV-vis spectrophotometer (Shimadzu, Tokyo, Japan) at 597 nm. Then the solid was treated with 2 μM ethanolamine to give Fe_3_O_4_@PDA/Hyp NSs. The final product was dispersed in ethanol for further use.

Fe_3_O_4_@PDA NSs were prepared and used as a control by following the same procedure as described for Fe_3_O_4_@PDA/Hyp NSs without the template.

### 2.4. Determination of Static Adsorption Capacity of Fe_3_O_4_@PDA/Hyp for Hypericin (Q)

To a centrifuge tube of 10 mL, 4 mg of Fe_3_O_4_@PDA/Hyp or Fe_3_O_4_@PDA NSs were added into a 4 mL known concentration of hypericin (59.4 μM unless specified) acetone solution. The tube was shaken at room temperature for 24 h (unless specified) in the dark. Then a magnet was used for separation, and the concentration of hypericin in the supernatant was measured with a UV-Vis spectrophotometer at 597 nm (Figures S6 and S7).

The adsorption capacity (*Q*, μg/g) of the NSs (Fe_3_O_4_@PDA/Hyp or Fe_3_O_4_@PDA) towards the test molecule was calculated by the following equation:

(1) $$Q=\frac{M(C_{0}-C_{e})V}{W}$$

where *C*_0_ and *C*_e_ represent the initial and equilibrium concentrations of the test molecule in acetone (μM), respectively; *M* is the molecular weight of the test molecule; *V* (L) is the volume of the solution, and *W* is the dry weight of the NSs (g).

The specific adsorption capacity of Fe_3_O_4_@PDA/Hyp NSs (*Q*_s_) towards hypericin is defined as the neat adsorption capacity of Fe_3_O_4_@PDA/Hyp over that of Fe_3_O_4_@PDA, and is calculated according to Equation (2):

(2) $$Q_{s}=Q_{1}-Q_{2}$$

where *Q*_1_ and *Q*_2_ are the static adsorption capacity of Fe_3_O_4_@PDA/Hyp and Fe_3_O_4_@PDA (μg/g) towards hypericin, respectively. 

### 2.5. Dynamic Adsorption Test

To investigate the adsorption kinetics of Fe_3_O_4_@PDA/Hyp (or Fe_3_O_4_@PDA) NSs, 4 mg of Fe_3_O_4_@PDA/Hyp (or Fe_3_O_4_@PDA) NSs were weighed into hypericin solution (59.4 μM, 4 mL) in a 10 mL centrifuge tube. The tubes were shaken at room temperature for the different time intervals (0.25, 1, 2, 4, 6, 8, and 12 h, respectively) in the dark. Then a magnet was used for the separation, and the concentration of hypericin in the supernatant was measured with a UV-Vis spectrophotometer at 597 nm.

### 2.6. Selectivity of Fe_3_O_4_@PDA/Hyp and Fe_3_O_4_@PDA for Hypericin

The binding selectivity of Fe_3_O_4_@PDA/Hyp and Fe_3_O_4_@PDA NSs was evaluated by measuring their binding capacities towards hypericin and two other molecules of protohypericin and emodin. 4 mg of the Fe_3_O_4_@PDA/Hyp or Fe_3_O_4_@PDA NSs were incubated respectively with 4 mL of hypericin, protohypericin, and emodin solution (59.4 μM in acetone) at 25 °C. After being incubated under continuously shaking for 24 h, the amounts of hypericin, protohypericin, and emodin bound to the Fe_3_O_4_@PDA/Hyp or Fe_3_O_4_@PDA NSs were measured, respectively. The binding selectivity of the NSs towards different molecules was compared using the “selectivity factor” (*SF*) and “imprinting factor” (*IF*) [24] that can be defined by the following equations:

(3) SF=Q1Q′1

where *Q*_1_’ is the adsorption capacity of the Fe_3_O_4_@PDA/Hyp NSs (μg/g) towards a non-template molecule.

(4) IF=QMIPQNIP

where *Q*_MIP_ and *Q*_NIP_ is the adsorption capacity of the Fe_3_O_4_@PDA/Hyp and Fe_3_O_4_@PDA NSs (μg/g) towards a test molecule, respectively.

### 2.7. Adsorption–Extraction Cycles

One adsorption–extraction cycle consisted of loading the template, reaching equilibrium adsorption, followed by the extraction of the template. For the adsorption, 4 mg of Fe_3_O_4_@PDA/Hyp was added to 4 mL 59.4 μM template in acetone. The suspension was incubated with a shaker at 25 °C for 8 h. Then the NSs were collected with a magnet and washed following the extraction procedure.

### 2.8. Preparation of the Herb Extract Solution

To prepare the herb extract, fresh flowers were picked up from *Hypericum perforatum* plant just before the extraction process. The extraction was performed in the dark. The procedure was as following: 10 g fresh flowers was charged to a 2-L beaker and immersed with distilled water at 50 °C for 2 h. Then the flowers were transferred to a 1000-mL flask and refluxed with 500 mL methanol-water mixture (80:20, *v*/*v*) for 6 h. The contents in the flask were cooled to room temperature and filtered. The filtrate was dried under reduced pressure. The residue was dissolved with acetone and filtered. The filtrate was combined and the volume was adjusted with a 25 mL volumetric flask.

### 2.9. HPLC Analysis

A total of 8 mL of the herb extract solution was mixed with 1 mL of hypericin and 1 mL of protohypericin solution (each with a concentration of 600 μM in acetone), to obtain an original solution for adsorption. To 4 mL of this final solution, 4 mg of the NSs (Fe_3_O_4_@PDA/Hyp or Fe_3_O_4_@PDA) was added. The mixture was shaken for 8 h. The supernatant was analyzed by HPLC (Shimadzu, Tokyo, Japan), with C18 reversed-phase column (5 μm, 4.6 mm × 150 mm) at 25 °C. The mobile phase consisted of 50% acetonitrile, 50% of the mixture of ammonium acetate-acetic acid buffer (0.3 M, pH = 6.96) and methanol (1:4, *v*/*v*); detection wavelength: 590 nm; flow rate: 0.4 mL/min; injection volume: 10 μL.

## 3. Results and Discussion

### 3.1. Synthesis of Fe_3_O_4_@PDA/Hyp

The synthesis of Fe_3_O_4_@PDA/Hyp is described in Scheme 1. Dopamine is a small molecule containing catechol and amino groups. As illustrated in Scheme 1a, when mixed with the template, dopamine formed assembly with hypericin via hydrogen bonding and π–π interaction. The stability of the assembly and hypericin under the reaction conditions was investigated by UV-Vis spectroscopy (see Figure S4). Comparing to the spectrum of hypericin in the mixture of Tris-HCl buffer/acetone without dopamine, only a blue shift was observed in the spectrum of hypericin with dopamine, even after the prolongation of time. The result indicated that both the assembly and hypericin were stable under the reaction conditions. The oxidization of dopamine under the reaction condition, alongside with its noncovalent self-assembly and covalent polymerization, led to a cross-linked PDA layer deposited on the surface of MNSs [20]. Removal of hypericin from the PDA layer gave Fe_3_O_4_@PDA/Hyp NSs.

### 3.2. Characterization of Fe_3_O_4_@PDA/Hyp

#### 3.2.1. FTIR Analysis

**Figure 1:**
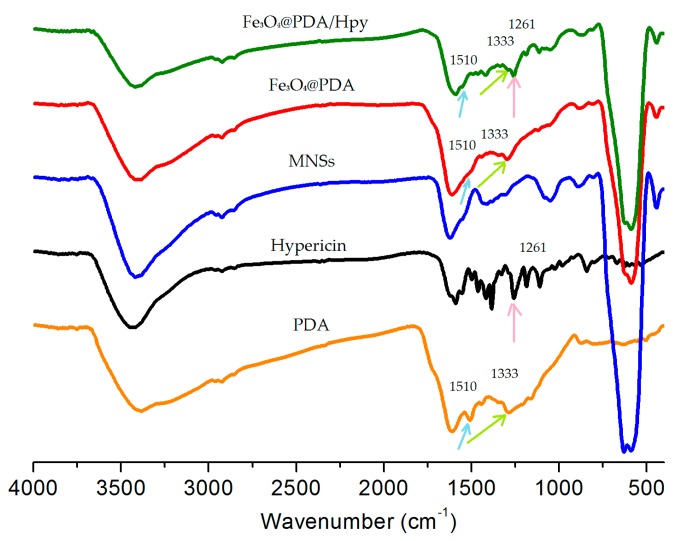
FTIR spectra of Fe_3_O_4_@PDA/Hyp, Fe_3_O_4_@PDA, MNSs, Hypericin and polydopamine (PDA).

To verify the self-polymerization of dopamine on the surface of MNSs in the absence or presence of hypericin, Fe_3_O_4_@PDA and Fe_3_O_4_@PDA/Hyp were analyzed by FTIR spectroscopy. As shown in Figure 1, two characteristic bands of 1510 and 1333 cm^−1^ from the spectrum of PDA, ascribed to C=N and C–N–C stretching vibration [25], respectively were found on both spectra of Fe_3_O_4_@PDA and Fe_3_O_4_@PDA/Hyp; This was also the case for the characteristic band of 1261 cm^−1^ (C–O stretching vibration) from the spectrum of hypericin on the spectrum of Fe_3_O_4_@PDA/Hyp. The results imply that dopamine successfully polymerized on the surface of MNSs with or without the presence of hypericin under the reaction conditions used in this work.

#### 3.2.2. TEM and DLS Analysis

**Figure 2:**
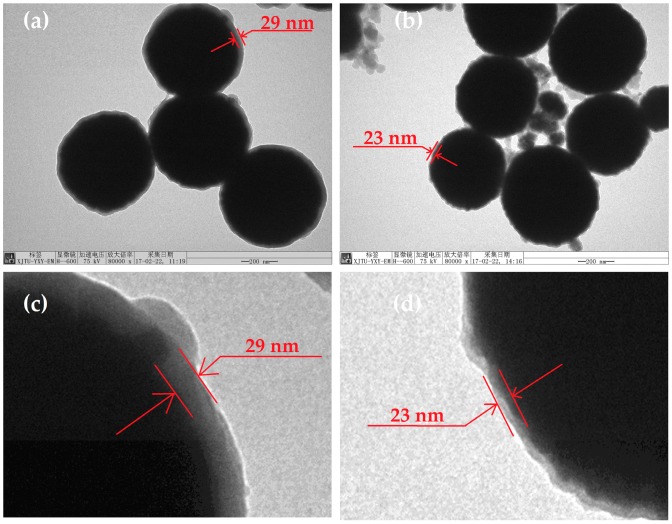
TEM images of the nanospheres (NSs) prepared: (**a**) Fe_3_O_4_@PDA NSs; (**b**) Fe_3_O_4_@PDA/Hyp NSs; (**c**,**d**) are the enlarged images corresponding to (**a**,**b**), respectively. Scale bar: 200 nm.

The morphology and the structure of Fe_3_O_4_@PDA/Hyp NSs were further studied by TEM and DLS. According to the TEM images (Figure 2), a thin layer around MNSs with a thickness of about 29 nm for Fe_3_O_4_@PDA NSs, and 23 nm for Fe_3_O_4_@PDA/Hyp NSs, was clearly seen. The thickness obtained in this work is similar to that reported in the literature [26]. The spherical shape of MNSs was preserved after polymerization. DLS analysis (Table 1 and Figure S5) showed the average diameter of Fe_3_O_4_/PDA and Fe_3_O_4_@PDA/Hyp NSs increased by 60 and 46 nm, to 573 and 559 nm, respectively, compared with that of MNSs (513 nm). The increased thickness in both cases coincided with the thickness of the PDA layer measured by TEM. 

In addition, when Fe_3_O_4_@PDA and Fe_3_O_4_@PDA/Hyp NSs were subjected to the extraction process, the ζ potential of Fe_3_O_4_@PDA/Hyp NSs became more negative (from −0.86 to −9.96), while that of Fe_3_O_4_@PDA NSs was less affected (see Table 1). The difference of the ζ potentials of Fe_3_O_4_@PDA/Hyp NSs before and after extracting process may be ascribed to the removal of the templates from Fe_3_O_4_@PDA/Hyp NSs. All these results indicate that the desired core-shell structure of Fe_3_O_4_@PDA/Hyp NSs can be prepared conveniently via dopamine, self-polymerized on the surface of MNSs in the presence of hypericin.

#### 3.2.3. BET Analysis

The average pore diameter, surface area and pore volume of the prepared Fe_3_O_4_@PDA/Hyp and Fe_3_O_4_@PDA NSs in this work were analyzed by BET method. The results are listed in Table 2. Although the average pore diameters of Fe_3_O_4_@PDA/Hyp and Fe_3_O_4_@PDA NSs were similar, the surface area and the pore volume of the Fe_3_O_4_@PDA/Hyp NSs were nearly 4.5 and 4 times, higher than those of the Fe_3_O_4_@PDA NSs, respectively. 

### 3.3. Optimization of Preparation Conditions for Specific Adsorption Capacity of Fe_3_O_4_@PDA/Hyp NSs

Considering that adsorption capacity of MIPs for targeting molecules can be affected by a number of parameters, the preparation conditions of Fe_3_O_4_@PDA/Hyp were screened and optimized, including monomer concentration, molar ratio of template to monomer, solvent, pH value, post-treatment, etc.

#### 3.3.1. Effect of Monomer Concentration on Specific Adsorption Capacity

**Figure 3:**
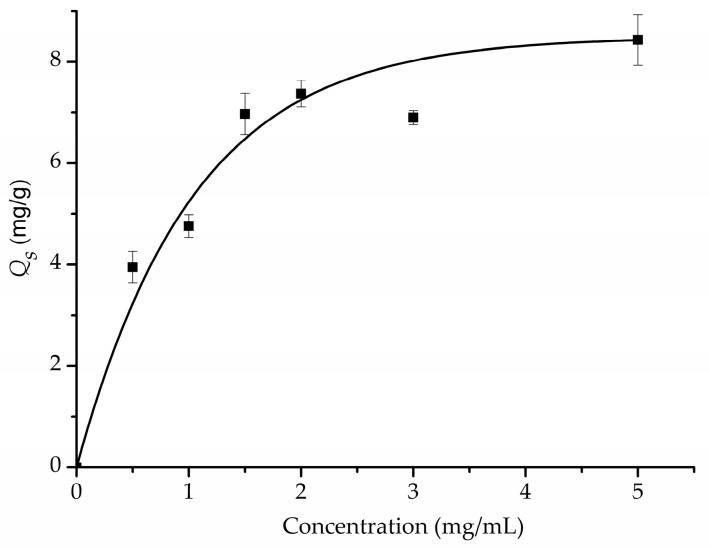
Effect of concentration of dopamine on specific absorption capacity (*Q*_s_).

For SMIP, the concentration of monomer is a key factor that affects the thickness of the polymeric layer and the number of the recognition sites formed. It has been suggested the concentration of dopamine should be at least 2 mg/mL to form a PDA film on the surface of substrate [20]. To evaluate the effect of dopamine concentration on specific adsorption capacity, a series of Fe_3_O_4_@PDA/Hyp NSs were prepared by varying the concentration of dopamine in a range of 0.5–5 mg/mL. As shown in Figure 3, *Q*_s_ increased gradually when the concentration of dopamine increased from 0.5 to 2 mg/mL, and reached a plateau with further increase of dopamine concentration. Interestingly, self-polymerization of dopamine in the system could take place either on the surface of the MNSs or in the solution. When the concentration of dopamine was lower than 2 mg/mL, self-polymerization of dopamine mainly took place on the surface of the MNSs, which led to the formation of a PDA layer around the MNSs. The higher the concentration, the thicker the layer, and therefore the more recognition sites led to a higher *Q*_s_ value. However, a concentration of dopamine higher than 2 mg/mL increased the opportunity of its self-polymerization in solution and led to the formation of pure PDA nanoparticles. Hence, we chose 2 mg/mL as the optimal concentration of dopamine for further study.

#### 3.3.2. Effect of the Ratio of Hypericin to Dopamine on Specific Adsorption Capacity

**Figure 4:**
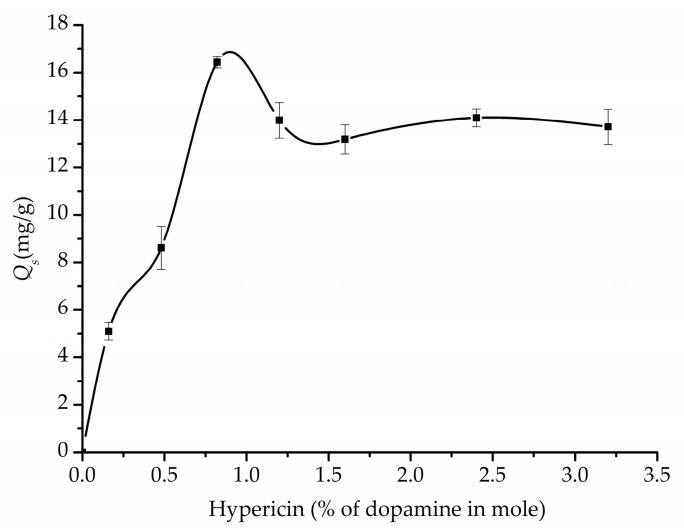
Effect of amount of hypericin (% of dopamine in mole) on specific absorption capacity (*Q*_s_).

The ratio of template to monomers (including comonomer) has long been recognized as one of the crucial parameters for selective adsorption capacity of MIPs. The optimal ratio can vary in a wide range. For example, a value of 1/155 was reported for a MIP towards EA9A [27], while in another system, 1/41 was used to prepare a MIP for protonated primary alkylamine [24]. The effect of the ratio of hypericin to dopamine (in mole percentage) on specific adsorption capacity was studied by varying the ratio between 0.16% and 3.2%. The results were summarized in Figure 4. It can be seen that the *Q*_s_ value increased with the ratio of hypericin to dopamine, and reached to a vertex at a ratio of 0.82% (equals to 1/122). Further increase of the ratio led to a slight decrease of the *Q*_s_. Therefore, the best ratio to acquire an optimal *Q*_s_ was determined to be 0.82%.

#### 3.3.3. Effect of the Amount of Acetone on Specific Adsorption Capacity

**Figure 5:**
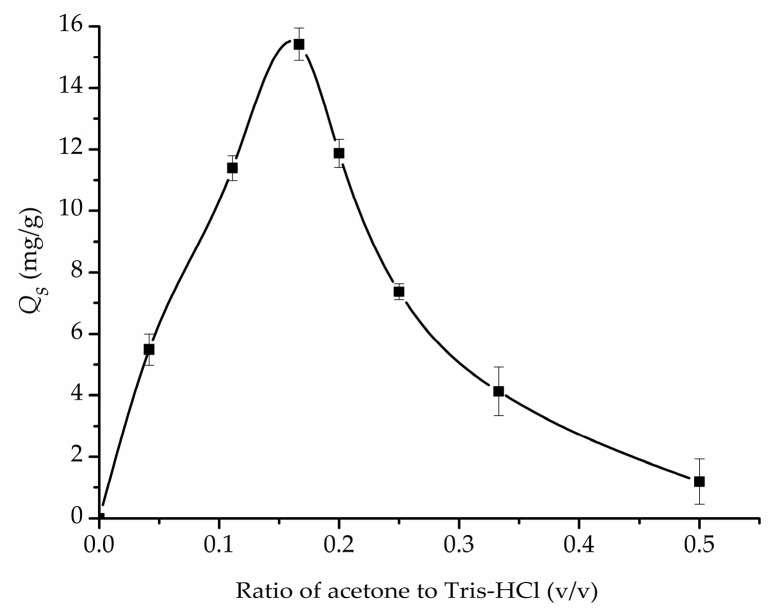
Effect of the ratio of acetone to Tris-HCl buffer (*v*/*v*) on specific adsorption capacity (*Q*_s_).

Considering hypericin is insoluble in water, it is necessary to dissolve it in a suitable organic solvent before the imprinting process. Acetone, as one of the few organic solvents which can dissolve hypericin and is miscible with water, was chosen and its influence on *Q*_s_ was investigated accordingly. As shown in Figure 5, the amount of acetone used during imprinting affected *Q*_s_ severely. The range of the ratio of acetone to Tris-HCl buffer (*v*/*v*) varied from 1/24 to 1/2. *Q_s_* was found to reach a maximum at the ratio of acetone to Tris-HCl around 1/6, and drop dramatically afterwards. It was deduced that acetone affected *Q*_s_ by altering the aggregation status of hypericin in the system and affecting the polymerization rate of dopamine and nanostructure of the polymer network.

#### 3.3.4. Effect of Other Parameters on Specific Adsorption Capacity 

Besides the parameters discussed above, some other parameters (such as pH, temperature, mixing order, post-treatment, etc.) greatly influence *Q*_s_, as shown in Table 3. As mentioned before, self-polymerization of dopamine is induced by oxygen in a weak alkaline solution; therefore, the effect of pH of Tris-HCl on *Q*_s_ at 7.5, 8.0 and 8.5 was explored. It was found that *Q*_s_ obtained at pH 8.0 is the highest within the pH range studied. Imprinting at a lower temperature resulted in the decrease of *Q*_s_, for instance from 16.40 mg/g at 25 °C to 10.12 mg/g at 0 °C. The change in the order of addition of hypericin and dopamine (i.e., mixing dopamine with MNSs before the addition of hypericin) led to a significant decrease of *Q*_s_, from 16.40 to 6.44 mg/g. Moreover, the post-treatment of Fe_3_O_4_@PDA/Hyp with ethanolamine nearly doubled the *Q*_s_, from 8.02 to 16.40 mg/g. The increase of *Q*_s_ might be ascribed to the deactivation of the surface of PDA by ethanolamine via Schiff base reaction and/or Michael addition [28].

Due to the before-mentioned results, we concluded that Fe_3_O_4_@PDA/Hyp NSs with an optimal *Q*_s_ value were prepared under the conditions used in Entry 2—i.e., 25 mg MNSs suspended in 41.67 mL Tris-HCl buffer solution was mixed with 2.7 mg hypericin dissolved in 8.33 mL acetone under mechanical stirring (acetone/Tris-HCl = 1/6 in volume, pH = 8.0) for 10 min, followed by the addition of 100 mg dopamine. The polymerization was run at room temperature in the atmosphere for 4 h. The nanospheres were subjected to the extracting process for removal of the template, followed by post-treatment with ethanolamine to obtain the best Fe_3_O_4_@PDA/Hyp NSs.

### 3.4. Dynamic Adsorption Study

**Figure 6:**
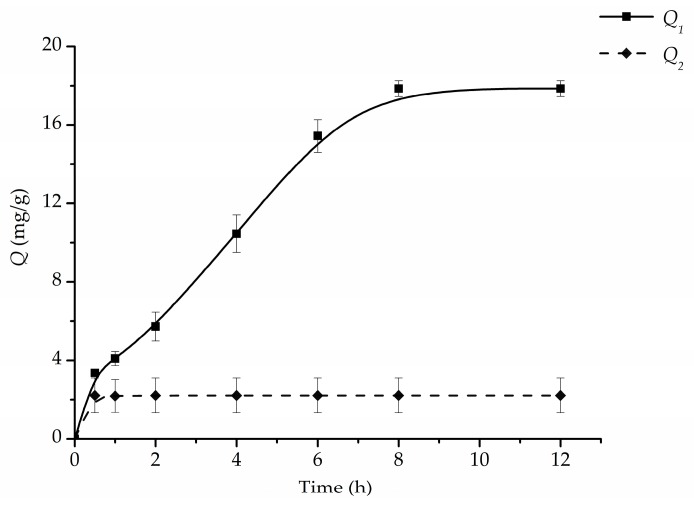
Dynamic adsorption of hypericin on Fe_3_O_4_@PDA/Hyp and Fe_3_O_4_@PDA NSs.

The equilibrium adsorption isotherms of Fe_3_O_4_@PDA/Hyp and Fe_3_O_4_@PDA NSs for the binding of hypericin were studied, and the results are shown in Figure 6. As observed in Figure 6, the adsorption process of the Fe_3_O_4_@PDA/Hyp NSs displayed two periods: the adsorption amount increased quickly and reached to about one third of the total adsorption capacity during the first hour. After this period, adsorption rate slowed down, and the equilibrium was achieved at 8 h. The explanation for this phenomenon is that the recognition sites located on the surface of PDA layer was ascribed to the binding of hypericin molecules in the first period, and then the diffusion of hypericin into the internal binding sites resulted in the slow adsorption rate, when the surface recognition sites became saturated. While the binding of Fe_3_O_4_@PDA towards hypericin saturated quickly after 0.25 h, the prolongation of contact time did not have a positive effect on the absorption. This may be explained by the much smaller pore volume and surface area of Fe_3_O_4_@PDA (proved by BET), where the non-specific absorption of hypericin mainly occurred on the surface of Fe_3_O_4_@PDA, and the mass transfer for hypericin into the interior of the PDA was inhibited. Compared to Fe_3_O_4_@PDA, a much stronger adsorption was observed on Fe_3_O_4_@PDA/Hyp NSs, which demonstrated the specific recognition sites were well-formed on the surface of Fe_3_O_4_@PDA/Hyp NSs.

### 3.5. Maximum of Specific Adsorption Capacity

To investigate the binding performance of Fe_3_O_4_@PDA/Hyp NSs prepared under the optimized conditions, binding experiments were conducted with different concentrations of hypericin, varying in a range of 0 to 100 μM. 

**Figure 7:**
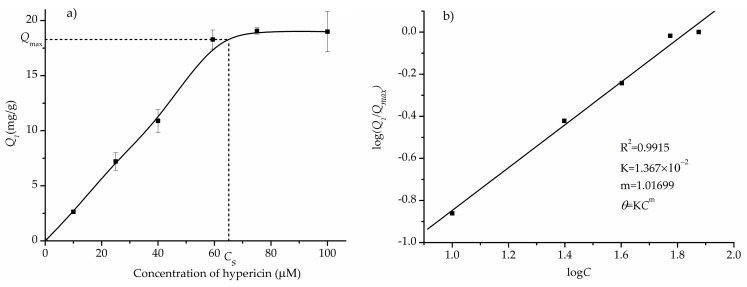
(**a**) The adsorption isotherm of Fe_3_O_4_@PDA/Hyp NSs towards hypericin; (**b**) The fitting plot of the adsorption isotherm of Fe_3_O_4_@PDA/Hyp NSs towards hypericin by Langmuir isotherm.

The adsorption isotherm of Fe_3_O_4_@PDA/Hyp NSs towards hypericin is shown in Figure 7a. It can be seen that *Q*_1_ increased with the concentration of hypericin before the saturation concentration *C*_S_ (59.4 μM), and reached a maximum adsorption at *C*_S_. Further increase in concentration did not cause any difference in adsorption process. Obviously, it fits Langmuir adsorption isotherm. The Langmuir equation is expressed as following:

(5) $$\theta =KC^{m}$$

where θ equals to *Q*_1_/*Q*_max_. The fitting plot is shown in Figure 7b. The fact that *m* value was close to 1 implied that the binding sites of Fe_3_O_4_@PDA/Hyps were homogeneous. The associated constant obtained was 1.367 × 10^−2^ M^−1^. The *R*^2^ value of 0.9915 showed a good consistency of the Langmuir equation with the measured data.

The specific adsorption of Fe_3_O_4_@PDA/Hyp NSs towards hypericin (*Q*_s_), as shown in Table 4, also increased with the concentration of hypericin, and achieved a maximum value of 16.30 mg/g at *C*_S_. It is worth mentioning that Fe_3_O_4_@PDA/Hyp NSs prepared under the optimized conditions in this work has a much higher specific absorption capacity for hypericin than that reported MIP via bulk polymerization (0.646 mg/g) [29]. 

### 3.6. Binding Selectivity

**Figure 8:**
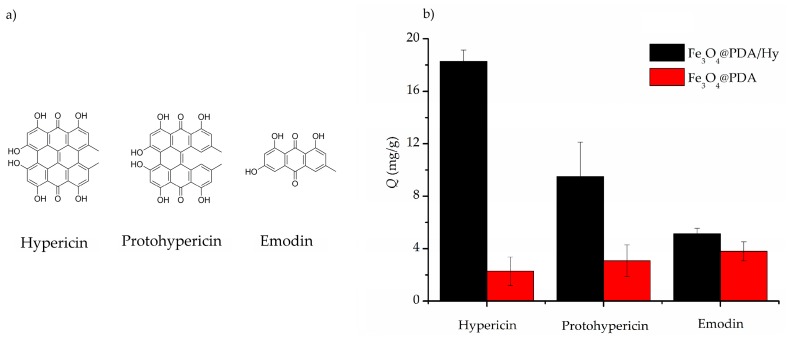
(**a**) The chemical structures of hypericin, protohypericin, and emodin; (**b**) Selective bindings of Fe_3_O_4_@PDA/Hyp (**black**) and Fe_3_O_4_@PDA (**red**) NSs toward to hypericin, protohypericin, and emodin, respectively.

In order to examine the selectivity of Fe_3_O_4_@PDA/Hyp NSs toward template hypericin, protohypericin and emodin were chosen as competitors of hypericin they are all quinone-type structures containing common hydroxyl groups, similar to hypericin. Fe_3_O_4_@PDA/Hyp or Fe_3_O_4_@PDA NSs were incubated respectively with the same amount of hypericin, protohypericin, and emodin under the same conditions. The adsorption capacity of Fe_3_O_4_@PDA/Hyp and Fe_3_O_4_@PDA NSs towards the three quinone compounds was summarized in Figure 8b. Fe_3_O_4_@PDA/Hyp NSs showed a higher adsorption for hypericin (18.2 mg/g) than that for protohypericin and emodin (9.4 and 5.1 mg/g, respectively). The binding selectivity of the polymer particles was evaluated with *SF* and *IF*, respectively. The results are listed in Table 5. *SF* of Fe_3_O_4_@PDA/Hyp NSs towards protohypericin (a structurally similar molecule) and emodin (a structurally dissimilar molecule), was 1.92 and 3.55, respectively. *IF* of Fe_3_O_4_@PDA/Hyp NSs towards hypericin, protohypericin, and emodin, was 8.01, 3.07, and 1.36, respectively. These indicated that Fe_3_O_4_@PDA/Hyp NSs had the best selectivity for hypericin among the three tested compounds. In addition, the *IF* value of Fe_3_O_4_@PDA/Hyp NSs towards hypericin in this work was much higher than that reported [29,30].

### 3.7. Reproducibility and Reusability Evaluation

**Figure 9:**
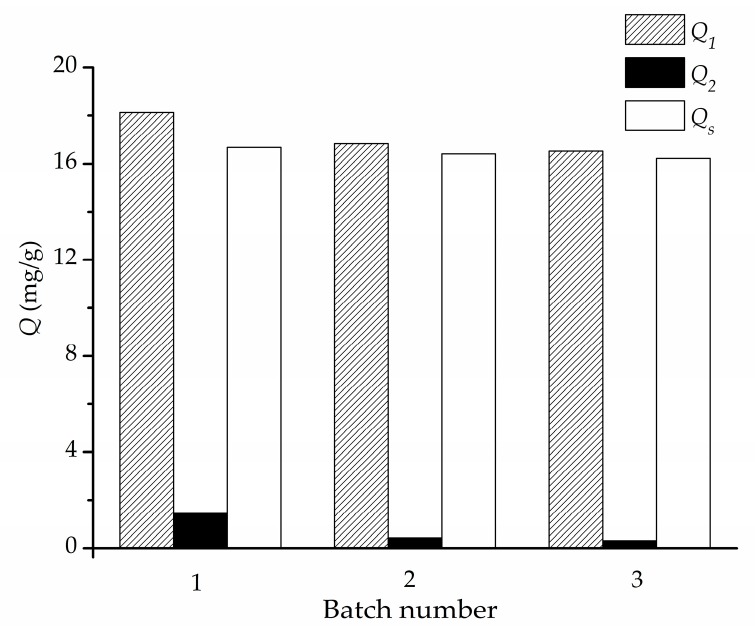
Reproducibility study of the approach for fabrication of Fe_3_O_4_@PDA/Hyp NSs under the optimized conditions.

The reproducibility of the approach for fabrication of Fe_3_O_4_@PDA/Hyp NSs was evaluated by three parallel experiments. As is shown in Figure 9, similar specific adsorption capacities of Fe_3_O_4_@PDA/Hyp NSs prepared individually at the optimal conditions were measured with 16.68, 16.41 and 16.22 mg/g, respectively. The results indicate that the approach established in this work for fabrication of core-shell Fe_3_O_4_@PDA/Hyp NSs for selective recognition of hypericin is reproducible.

The reusability of MIPs is crucial in developing applications that are reliable, economic and sustainable [31]. Unfortunately, the evaluation on the Fe_3_O_4_@PDA/Hyp NSs prepared in this work showed that their reusability is not optimal. As shown in Figure S8, the absorption capacity decreased by 35% at the second cycle, and ~50% at the fourth cycle.

### 3.8. Adsorption of Fe_3_O_4_@PDA/Hyp NSs from the Herb Extract

**Figure 10:**
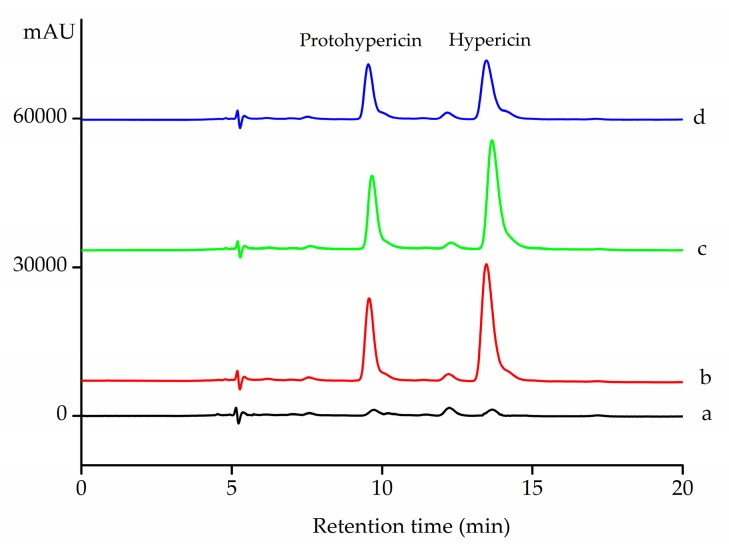
HPLC chromatograms. (**a**) the herb extract; (**b**) the mixture of the extract, hypericin and protohypericin before adsorption; (**c**) the supernatant after the adsorption of Fe_3_O_4_@PDA NSs; (**d**) the supernatant after the adsorption of Fe_3_O_4_@PDA/Hyp NSs.

To test the selective adsorption ability of Fe_3_O_4_@PDA/Hyp NSs towards hypericin in a real sample, a herb extract was prepared and mixed with equal amount of hypericin and protohypericin. After adsorption, the supernatant was analyzed by HPLC, and the results are shown in Figure 10 and Table 6. According to the HPLC chromatogram of the herb extract, both peaks of hypericin and protohypericin were found (Figure 10a) and were well-separated under the LC conditions used for the analysis. From Table 6, it can be seen that 47.8% of hypericin in the initial sample was absorbed by Fe_3_O_4_@PDA/Hyp, while only 8.3% was absorbed by Fe_3_O_4_@PDA. Compared to Fe_3_O_4_@PDA, Fe_3_O_4_@PDA/Hyp showed nearly 6 times higher adsorption toward hypericin. On the other hand, Fe_3_O_4_@PDA gave similar adsorption to both hypericin and protohypericin (8.3% and 11.5%, respectively), which implied that the adsorption was non-specific.

## 4. Conclusions

This work has for the first time established a facile approach to fabricate core-shell magnetic molecularly imprinted nanospheres for selective recognition of hypericin (Fe_3_O_4_@PDA/Hyp) NSs via self-polymerization of dopamine on the surface of MNSs. The core-shell structure of the synthesized Fe_3_O_4_@PDA/Hyp NSs was confirmed by TEM. The reaction conditions for adsorption capacity were screened, which revealed that the ratios of acetone to Tris-HCl buffer and hypericin to dopamine, and post-treatment with ethanolamine, are crucial in maximizing the imprinting efficiency. Fe_3_O_4_@PDA/Hyp NSs prepared under the optimized conditions demonstrate a high adsorption capacity (*Q* = 18.28 mg/g), and possess good binding selectivity towards hypericn. In addition, the approach established in this work has good reproducibility for fabrication of Fe_3_O_4_@PDA/Hyp.

## Figures and Tables

**Table 1:** The DLS data of the NSs prepared.

NSs	Paticle size (nm)	Polydispersity index	ζ Potential (mV)
MNSs	513 ± 98	0.444	1.35 ± 0.98
Fe_3_O_4_@PDA	572 ± 85	0.761	−3.96 ± 0.87
Fe_3_O_4_@PDA/Hyp	559 ± 40	0.385	−0.86 ± 0.08
Fe_3_O_4_@PDA after extracting process	574 ± 40	0.374	−1.38 ± 0.42
Fe_3_O_4_@PDA/Hyp after extracting process	561 ± 98	0.338	−9.96 ± 0.66

**Table 2:** The pore size, surface area, and the pore volume of the NSs.

NSs	Average pore diameter (nm)	Surface area (m^2^/g)	Pore volume (cm^3^/g)
Fe_3_O_4_@PDA/Hyp	8.095 ± 0.409	54.764 ± 4.189	0.111 ± 0.003
Fe_3_O_4_@PDA	9.185 ± 1.086	12.176 ± 2.166	0.028 ± 0.001

**Table 3:** *Q*_s_ of Fe_3_O_4_@PDA/Hyp NSs synthesized with different conditions ^a^.

Entry	pH	Temperature (°C)	*Q*_s_ (mg/g)	RSD
1	7.5	25	15.23 ± 0.08	0.005
2	8.0	25	16.40 ± 0.23	0.014
3	8.5	25	15.41 ± 0.52	0.034
4	8.0	0	10.12 ± 0.89	0.088
5 ^b^	8.0	25	6.44 ± 0.19	0.029
6 ^c^	8.0	25	8.02 ± 0.51	0.005

^a^ A total of 25 mg MNSs suspension in Tris-HCl were mixed with 2.7 mg hypericin in acetone under mechanical stirring in 50 mL solvent (acetone/Tris-HCl = 1/6 in volume) for 10 min, followed by the addition of 100 mg dopamine; ^b^ 25 mg MNSs suspension in Tris-HCl were mixed with 100 mg dopamine under mechanical stirring for 10 min, followed by the addition of 2.7 mg hypericin dissolved in acetone (total volume is 50 mL, acetone/Tris-HCl = 1/6 in volume); ^c^ Fe_3_O_4_@PDA/Hyp prepared according to Entry 2, but without post-treatment with ethanolamine.

**Table 4:** The effect of the concentration of hypericin on *Q*_s_.

Concentration of hypericin (μM)	*Q*_1_ (mg/g)	RSD	*Q*_s_ (mg/g)	RSD
0	0	0	0	0
10	2.62 ± 0.03	0.006	0.92 ± 0.80	0.814
25	7.21 ± 0.71	0.110	3.72 ± 0.68	0.067
40	10.89 ± 0.71	0.095	8.10 ± 0.27	0.031
59.4	18.28 ± 0.73	0.047	16.30 ± 0.12	0.007
75	19.06 ± 0.02	0.015	10.58 ± 1.11	0.105
100	19.00 ± 0.04	0.096	14.96 ± 1.32	0.077

**Table 5:** The values of *SF* and *IF*.

Factor	Hypericin	Protohypericin	Emodin
*SF*	/	1.92	3.55
*IF*	8.01 (3.56 ^a^, 4.5 ^b^)	3.07	1.36

^a^ The value was from Ref. [29]; ^b^ The value was from Ref. [30].

**Table 6:** The adsorption capacity of hypericin for hyperforin perforatum.

Sample	Peak area (Hyp)	Peak area (Protohyp)	Adsorption of Hyp (%)	Adsorption of Protohyp (%)
Initial	676,506.3	364,871.0	/	/
Fe_3_O_4_@PDA	620,591.5	322,926.9	8.3	11.5
Fe_3_O_4_@PDA/Hyp	353,391.0	246,750.0	47.8	32.4

## Supplementary Materials

The following are available online at www.mdpi.com/2073-4360/9/4/135/s1. Figure S1: The ^1^H-NMR spectrum of emodin anthrone; Figure S2: The ^1^H-NMR spectrum of protohypericin; Figure S3: The ^1^H-NMR spectrum of hypericin; Figure S4: UV-Vis spectra of hypericin in Tris-HCl and acetone (*v*/*v* = 6:1, pH = 8.0) with or without the presence of dopamine; Figure S5: DLS histograms of MNSs and Fe_3_O_4_@PDA and magnetic response of MNSs; Figure S6: (a) UV-Vis spetra of hypericin, protohypericin and emodin, respectively, (b–d) Standard curves of hypericin, protohypericin and emodin; Figure S7: TEM images of MNSs (a) and their response to magnet in 10 s (b,c); Figure S8: Reusability study of Fe_3_O_4_@PDA/Hyp NSs.
