# Supplementary material for: A Facile Approach for Fabrication of Core-Shell Magnetic Molecularly Imprinted Nanospheres towards Hypericin
Source: Polymers (Basel). 2017 Apr 7;9(4):135. doi: 10.3390/polym9040135 (PMC6431851; doi:10.3390/polym9040135)
Supplement: Supplementary file 1 [file polymers-09-00135-s001.pdf]

Supplementary Materials:
A facile approach for fabrication of core-shell magnetic molecularly imprinted nanospheres toward hypericin

Wenxia Cheng, Fengfeng Fan, Ying Zhang, Zhichao Pei, Wenji Wang, and Yuxin Pei*

Shanxi Key Laboratory of Natural Products & Chemical Biology, College of Chemistry & Pharmacy, Northwest A&F University, Yangling, Shaanxi, China;

E-mail: peyx@nwafu.edu.cn; Fax: +86-29-87092769; Tel: +86-29-87091196

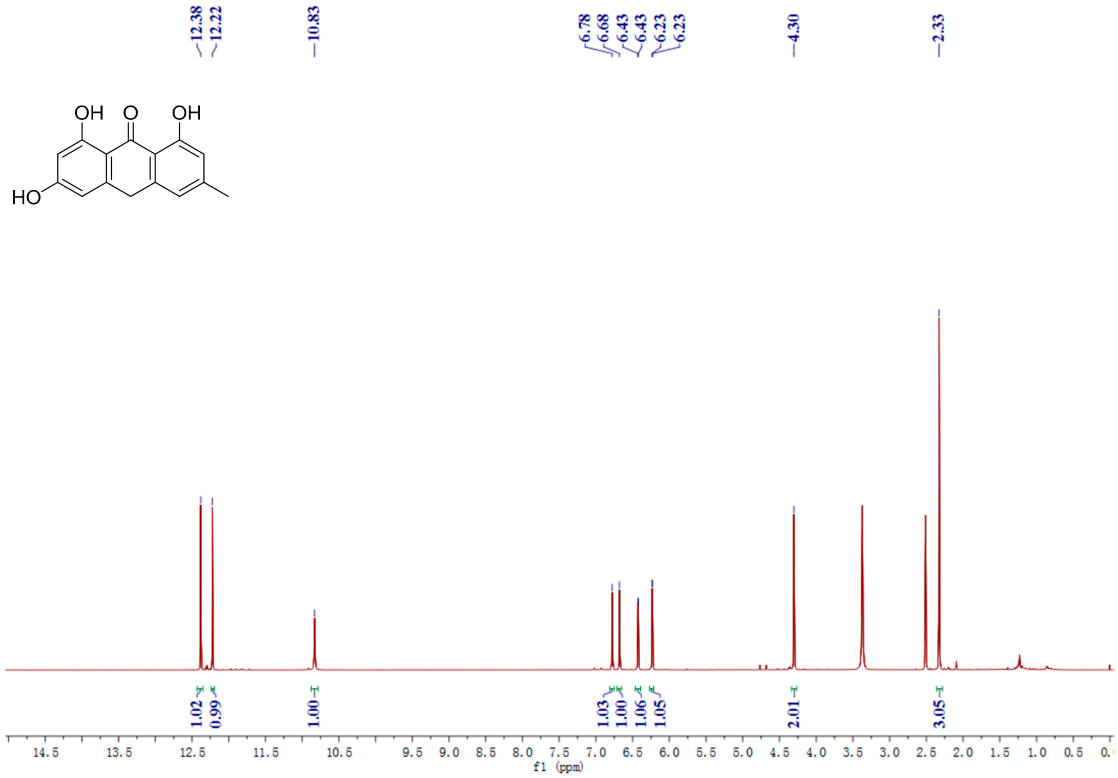

Figure S1. The ^{1}H-NMR spectrum of emodin anthrone.

Emodin anthrone: ^{1}H-NMR (500 MHz, DMSO-d_6) δ 12.38 (s, 1H), 12.22 (s, 1H), 10.83 (s, 1H), 6.78 (s, 1H), 6.68 (s, 1H), 6.44 (d, *J* = 1.8 Hz, 1H), 6.24 (d, *J* = 2.1 Hz, 1H), 4.30 (s, 2H), 2.33 (s, 3H).

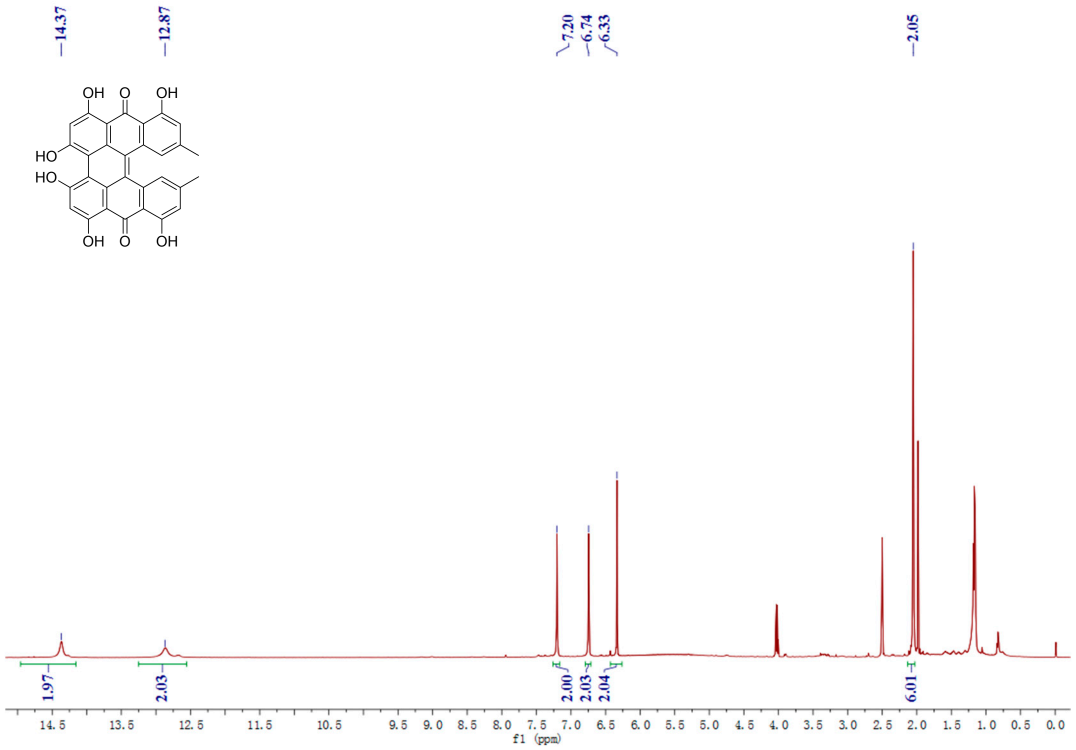

Figure S2. The ^{1}H-NMR spectrum of protohypericin.

Protohypericin: ^{1}H-NMR (500 MHz, DMSO-d_6) δ 14.37 (s, 2H), 12.87 (s, 2H), 7.20 (s, 2H), 6.74 (s, 2H), 6.33 (s, 2H), 2.06 (s, 6H).

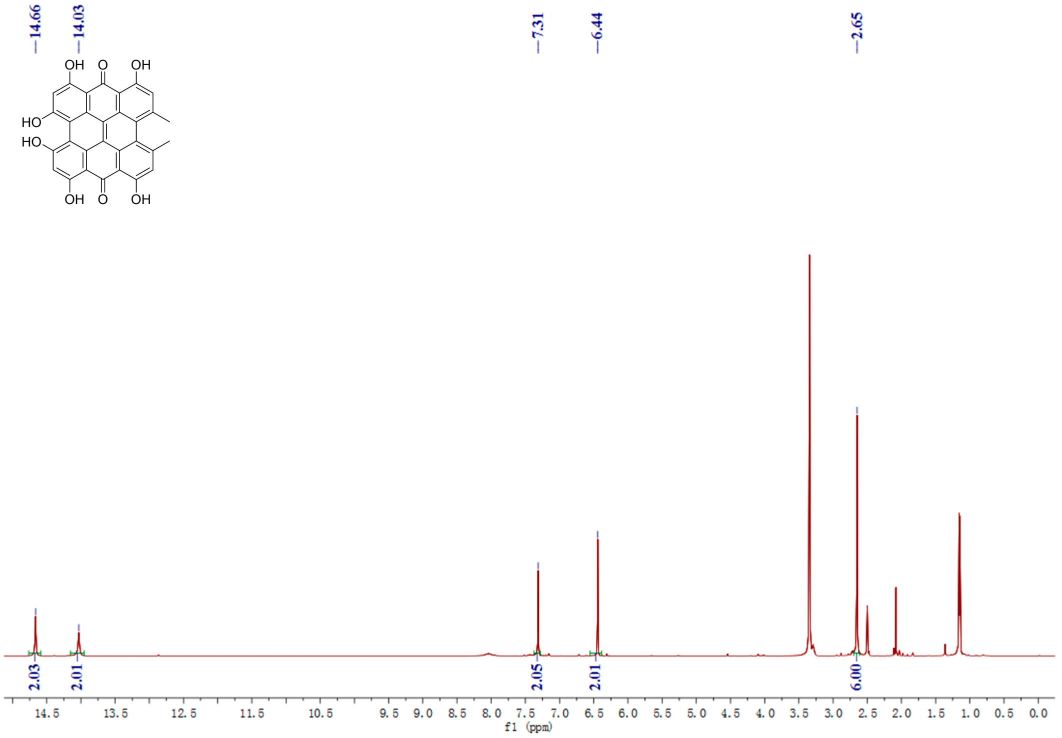

Figure S3. The ^{1}H-NMR spectrum of hypericin.

Hypericin: ^{1}H-NMR (500 MHz, CD_3OD) δ 14.66 (s, 2H), 14.03 (s, 2H), 7.31 (s, 2H), 6.44 (s, 2H), 2.65 (s, 6H).

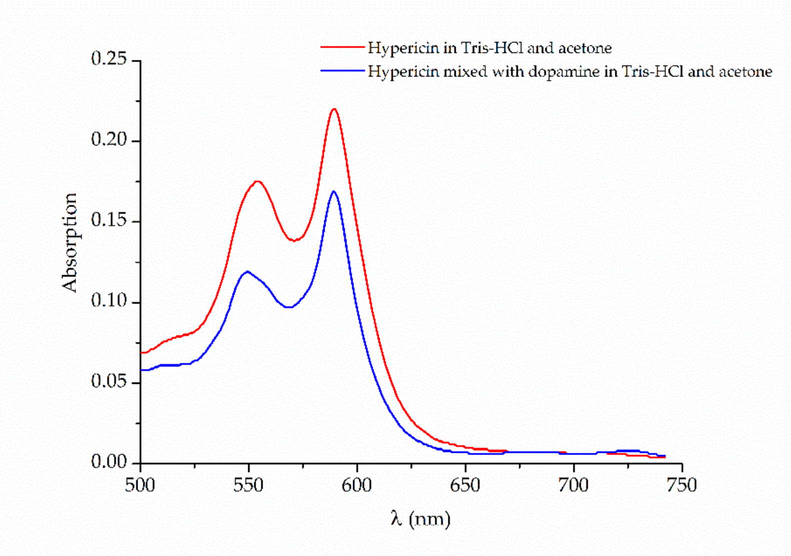

Figure S4. UV-Vis spectra of hypericin in Tris-HCl and acetone (v/v = 6:1, pH = 8.0) with (blue line), or without (red line) the presence of dopamine (2 mg/mL).

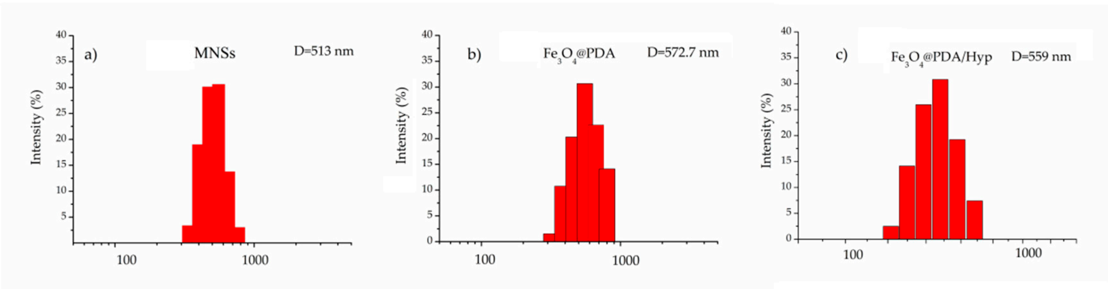

Figure S5. (a) DLS histogram of MNSs, PDI = 0.44; (b) DLS histogram of Fe_3O_4@PDA, PDI = 0.761; (c) DLS histogram of Fe_3O_4@PDA/Hyp, PDI = 0.385.

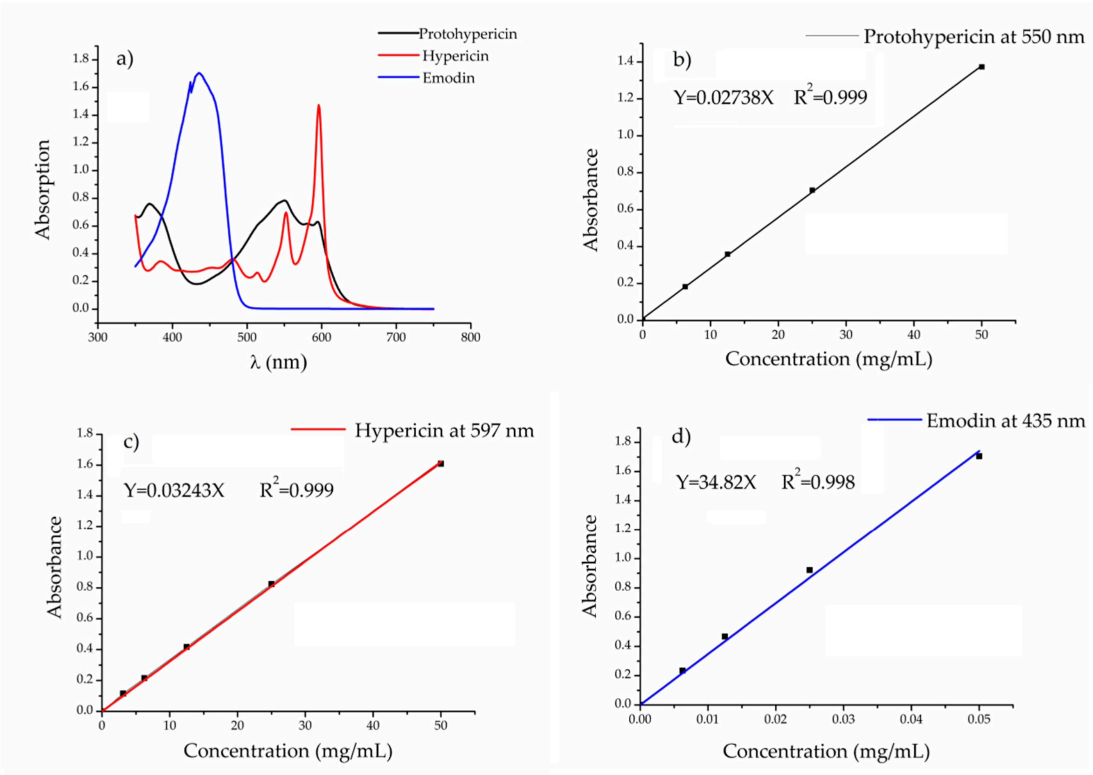

Figure S6. (a) UV-Vis spetra of hypericin, protohypericin, and emodin, respectively. (b) Standard curve of hypericin in acetone by UV-spectrophotometer at 597 nm. (c) Standard curve of protohypericin in acetone by UV-spectrophotometer at 550 nm. (d) Standard curve of emodin in acetone by UV-spectrophotometer at 435 nm.

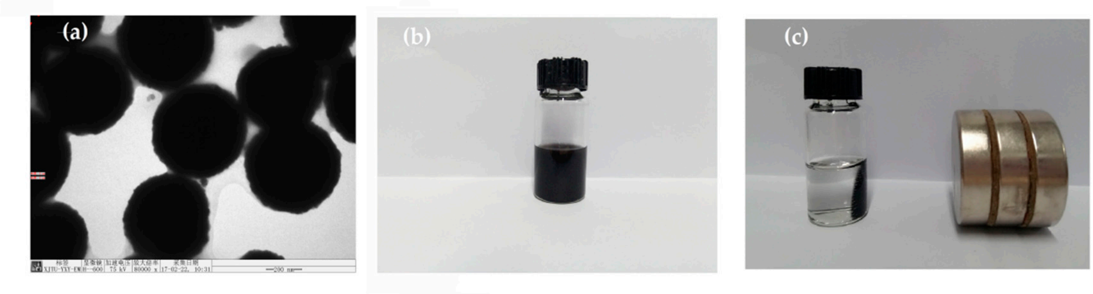

Figure S7. TEM images of MNSs (a) and their response to magnet in 10 s (b and c). Scale bar: 200 nm.

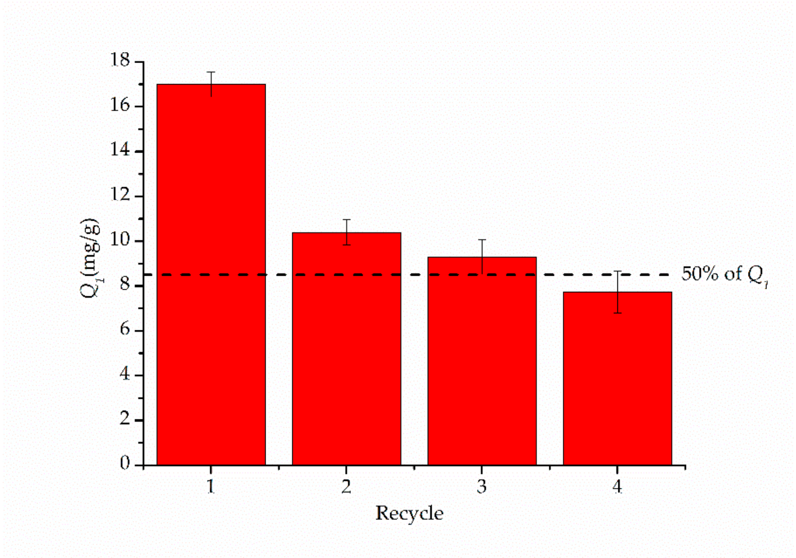

Figure S8. Reusability study of Fe_3O_4@PDA/Hyp NSs.
